# Mechanisms of social regulation change across colony development in an ant

**DOI:** 10.1186/1471-2148-10-328

**Published:** 2010-10-27

**Authors:** Dani Moore, Jürgen Liebig

**Affiliations:** 1 School of Life Sciences, Arizona State University, Tempe, USA

## Abstract

**Background:**

Mutual policing is an important mechanism for reducing conflict in cooperative groups. In societies of ants, bees, and wasps, mutual policing of worker reproduction can evolve when workers are more closely related to the queen's sons than to the sons of workers or when the costs of worker reproduction lower the inclusive fitness of workers. During colony growth, relatedness within the colony remains the same, but the costs of worker reproduction may change. The costs of worker reproduction are predicted to be greatest in incipient colonies. If the costs associated with worker reproduction outweigh the individual direct benefits to workers, policing mechanisms as found in larger colonies may be absent in incipient colonies.

**Results:**

We investigated policing behaviour across colony growth in the ant *Camponotus floridanus*. In large colonies of this species, worker reproduction is policed by the destruction of worker-laid eggs. We found workers from incipient colonies do not exhibit policing behaviour, and instead tolerate all conspecific eggs. The change in policing behaviour is consistent with changes in egg surface hydrocarbons, which provide the informational basis for policing; eggs laid by queens from incipient colonies lack the characteristic hydrocarbons on the surface of eggs laid by queens from large colonies, making them chemically indistinguishable from worker-laid eggs. We also tested the response to fertility information in the context of queen tolerance. Workers from incipient colonies attacked foreign queens from large colonies; whereas workers from large colonies tolerated such queens. Workers from both incipient and large colonies attacked foreign queens from incipient colonies.

**Conclusions:**

Our results provide novel insights into the regulation of worker reproduction in social insects at both the proximate and ultimate levels. At the proximate level, our results show that mechanisms of social regulation, such as the response to fertility signals, change dramatically over a colony's life cycle. At the ultimate level, our results emphasize the importance of factors besides relatedness in predicting the level of conflict within a colony. Our results also suggest policing may not be an important regulatory force at every stage of colony development. Changes relating to the life cycle of the colony are sufficient to account for major differences in social regulation in an insect colony. Mechanisms of conflict mediation observed in one phase of a social group's development cannot be generalized to all stages.

## Background

Kin selection theory can explain the evolution of cooperation within groups of related individuals, but unless group members are clones, there is also potential for conflict [1]. Because relatedness establishes the basis for cooperation and conflict within family groups, it has overshadowed other factors that affect the degree of conflict [2-4]. Variations in relatedness are not necessarily the most important force determining the extent of conflict in a social group; costs and benefits of altruism can be the dominant predictors of social behaviour [2,5]. In cooperative groups with a predetermined life cycle, such as a social insect colony, the group's developmental stage is one factor that may affect the degree of conflict within the group [6].

In the social Hymenoptera (ants, bees, and wasps), conflict may exist over male production [7]. In most species, workers retain functional ovaries and are capable of laying viable, male-destined eggs. Because workers are more closely related to their own sons than the queen's sons, workers are predicted to prefer producing their own sons over rearing the queen's sons. In reality, male production by workers is often absent or repressed under queenright conditions [4,8,9]. Worker sterility is hypothesized to be enforced by mutual policing. Worker policing occurs in two forms: (1) physical policing, in which workers with activated ovaries are attacked by nestmates [10-13], and (2) egg policing, in which workers detect and destroy worker-laid eggs [14-20]. The importance of policing in maintaining worker sterility has been emphasized in several recent papers [21-26].

Mutual worker policing can evolve if workers are more closely related to the queen's sons than to the sons of other workers (relatedness hypothesis). This is the case in polygynous or polyandrous species [14,16,27,28], but policing behaviour has also been described in species that are monogynous and monoandrous [19,29-31], and even in a clonal species [13]. In these species, workers are more related to other worker's sons than the sons of the queen, and thus worker policing cannot be explained solely on the grounds of relatedness. Instead, policing behaviour may have evolved in these species because the costs of worker reproduction reduce the inclusive fitness of workers (cost hypothesis) [4,32,33].

The costs of worker reproduction are not constant across colony development [5,6]. Incipient social insect colonies undergo a period of ergonomic growth in which new workers are added to the colony but no sexuals are produced [34]. Presumably, this reflects the relative value of colony growth over reproduction in the early stages of a colony's development. Worker reproduction is necessarily an investment in reproduction because worker-laid eggs can only develop into males, which do not work [35]. Worker reproduction further undermines ergonomic growth because reproductive workers are less productive than their non-reproductive counterparts [4,32,33,36-38]. Loss of worker productivity is especially costly to incipient colonies because the relative contribution of each worker is greatest when the colony is small [5], and colony mortality is highest in incipient colonies [39]. Because worker reproduction is more costly in incipient colonies than large colonies, worker policing is predicted to be strongest when the colony is young [6]. However, workers from incipient colonies have less incentive to reproduce, so the policing mechanisms present in large colonies may not be the same as in incipient colonies.

Effective policing requires that workers be able to identify cheaters or their eggs. Much evidence indicates hydrocarbons on the cuticles of adults or on the surfaces of eggs provide the information workers use to recognize the presence of accepted reproductives and to target reproductive cheaters. Hydrocarbons correlate reliably with fertility in more than 28 genera of ants, wasps, bees, and termites [40-44]. Physical policing can occur when reproductive workers exhibit fertility-related hydrocarbons on their cuticle [12,45-47]. Egg policing can occur when the surface hydrocarbons of worker-laid eggs lack the fertility-related hydrocarbons present on the eggs of the queen [18,19,48].

Fertility information may not be available as an informational basis for policing in incipient colonies. As predicted by the hypothesis that hydrocarbons are an honest indicator of reproductive capacity [40,49], the concentration of fertility compounds on both the cuticle and egg surface increases with egg-laying rate [50-52]. Queen egg-laying rate is positively correlated with colony size; queens of small colonies lay very few eggs per day [52-54]. Therefore, founding queens and their eggs are expected to lack the hydrocarbons characteristic of highly productive reproductives. This prediction has been tested in two species, *Camponotus floridanus *[52] and *Lasius niger*[55]. As predicted, the abundance of fertility-related hydrocarbons on a queen's cuticle increases as the colony grows and the queen becomes more productive. Incipient queens of *C. floridanus *lack the shorter-chained compounds present on the cuticles of established queens. Eggs laid by founding queens are chemically indistinguishable from worker-laid eggs, and workers from large colonies destroy incipient-queen-laid eggs as frequently as worker-laid eggs [52]. This presents an interesting discrepancy. Worker policing is theoretically predicted to be strongest in growing colonies, but the informational basis for policing used in large colonies is not available in incipient colonies.

We explore worker-policing behaviour across colony development. Because relatedness within a colony is constant across development, variations in relatedness cannot explain any change in policing behaviour we observe between incipient and large colonies. We also address the proximate mechanisms of worker policing to understand how workers accommodate the changes in fertility signalling that accompany colony growth.

Fertility signalling and worker policing have been studied extensively in the monogynous carpenter ant *C. floridanus*[19,38,52], in which queens are only single-mated [56]. In this species, qualitative and quantitative differences exist between the cuticular hydrocarbons of workers and established queens. Approximately half of the total amount of hydrocarbons present on an established queen's cuticle represent compounds that correlate with fertility [19,52]. Queens bearing these fertility-related hydrocarbons can be transferred between established colonies without aggression [57]. The eggs of established queens are coated with a blend of hydrocarbons similar to the hydrocarbons on the queen's cuticle [19,52]. Worker-laid eggs lack the shorter-chained, fertility-related hydrocarbons present on queen-laid eggs. When worker-laid eggs are introduced into a large, queenright colony, they are destroyed. Worker-laid eggs coated in queen hydrocarbons are destroyed less often than unmanipulated eggs, strongly suggesting hydrocarbons are responsible for the recognition of queen- and worker-laid eggs [19]. Physical policing does not occur in *C. floridanus *[38].

In this study, we tested the egg-policing behaviour of *C. floridanus *workers at three points in colony development. At each of the three points, we tested the response of workers to eggs laid by their own queen, eggs laid by an established queen (i.e., a queen at least one year of age with a colony of more than 1000 workers), and worker-laid eggs. We collected egg surface hydrocarbon data to correlate our behavioural results with the availability of relevant fertility information. In a second experiment, we explored the response to fertility information further by contrasting the response of workers from small colonies and large colonies to the introduction of foreign queens.

## Results

**Figure 1:**
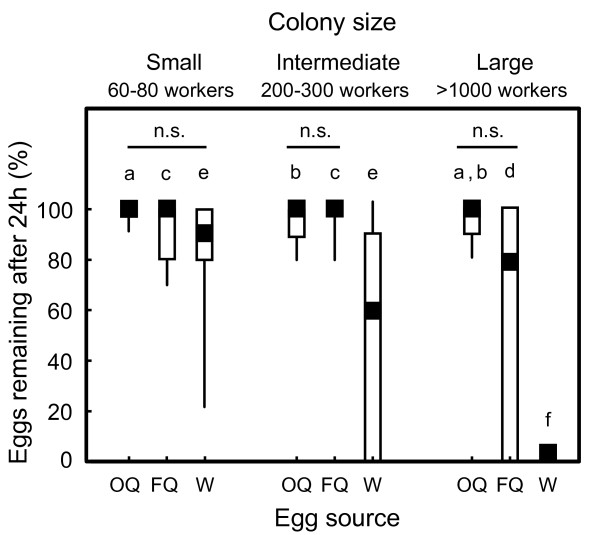
**Percentage of eggs recovered after 24 hours**. Egg-policing behaviour changed dramatically as colonies grew. Workers from small colonies tolerated eggs laid by their own queen (OQ), eggs laid by a foreign, established queen (FQ), and eggs laid by workers (W), whereas workers from large colonies destroyed worker-laid eggs. Points, boxes, and whiskers represent medians, quartiles, and ranges, respectively. Horizontal bars indicate no difference in the survival of eggs from different sources within a size class at α = 0.05; letters indicate differences in survival of eggs from a given source between size classes at α = 0.05. Randomized multi-factor ANOVA, *N *= 15, *F *= 13.74, p_10,000 _< 0.0001.

The egg-policing behaviour of *C. floridanus *workers changed dramatically during colony growth (Randomized multi-factor ANOVA, F = 13.74, p_10,000 _< 0.0001, Table 1, Figure 1). In small colonies (60-80 workers), workers tolerated all eggs, regardless of origin. There was no significant difference in the median percentage of eggs recovered after 24 hours from workers receiving eggs laid by their own queen (median = 100%, range = 90--100%, *N *= 15), an established queen (median = 100%, range = 70--100%, *N *= 15), or foreign workers (median = 90%, range = 20--100%, *N *= 15). Egg-policing behaviour emerged only with colony growth. In large colonies (>1000 workers), the percentage of eggs surviving the 24-hour discrimination assay remained high for workers receiving their own queen eggs (median = 100%, range = 80--100%, *N *= 15), but dropped to zero for eggs laid by workers (median = 0%, range = 0%, *N *= 15). Acceptance of eggs laid by foreign, established queens by workers from large colonies was highly variable (median = 60%, range = 0--100%, *N *= 15).

**Table 1:** Randomized multi-factor ANOVA with colony size and egg source as fixed factors and colony as a random factor

source	ss	df	ms	F	**p**_**10,000**_
colony	49.526	14	3.538	0.80	0.6711
colony size	387.393	2	193.696	43.61	< 0.0001
egg source	623.348	2	311.674	70.18	< 0.0001
colony size × egg source	282.074	4	70.519	15.88	< 0.0001
error	497.407	112	4.441		

**Figure 2:**
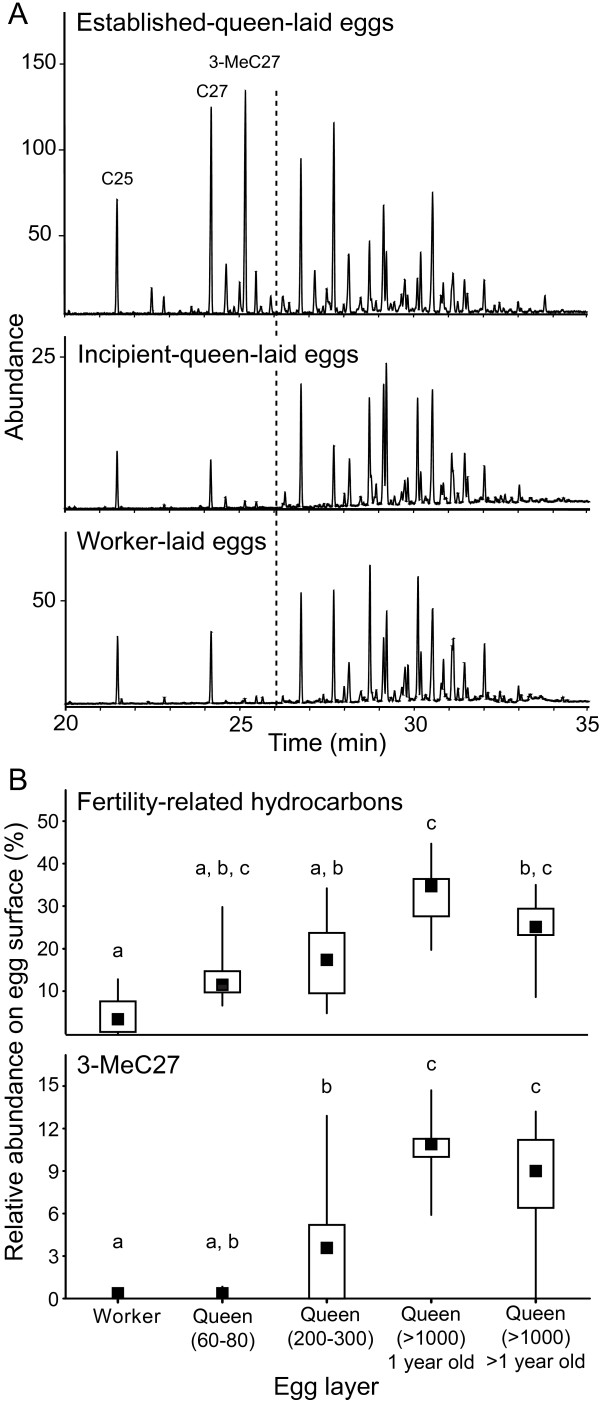
**Differences in egg surface hydrocarbons**. (A) Representative chromatograms showing the egg surface hydrocarbons on eggs laid by an established queen, eggs laid by an incipient queen, and eggs laid by workers. The shorter-chained compounds involved in fertility signalling appear to the left of the dotted line. The peak representing 3-methylheptacosane (3-MeC27), the most prominent compound in the fertility signal, is labelled. Also labelled are the peaks representing straight-chain alkanes *n-*pentacosane (C25) and *n-*heptacosane (C27). Additional compound identities are published in [19]. (B) The percentage of egg surface hydrocarbons represented by all shorter-chained, fertility-related compounds (top) and 3-methylheptacosane (bottom). Points, boxes, and whiskers represent medians, quartiles, and ranges, respectively. Letters indicate significant pairwise differences between eggs from different sources at α = 0.05. All shorter-chained, fertility-related hydrocarbons: median test, χ^2 ^= 30.2077, p < 0.0001, d.f. = 4. 3-Methylheptacosane: median test, χ^2 ^= 41.9077, *p *< 0.0001, d.f. = 4.

The proportion of fertility-related compounds on the surface of queen eggs increased with colony size, as reported in Endler *et al. *[52]. Shorter-chained, fertility-related compounds (*n*-pentacosane to 10-methyl-, 12-methyl-, 14-methyloctacosane) comprised a greater percentage of surface hydrocarbons on eggs laid by queens of large experimental colonies (median = 34.7%, range = 19.7--44.7%, *N *= 13), than by established-queen-egg donors (median = 25.1%, range = 8.6--35.0%, *N *= 15), intermediate queens (median = 16.6%, range = 4.7--34.2%, *N *= 12), incipient queens (median = 11.5%, range = 6.6--29.8%, *N *= 8), and workers (median = 3.4%, range = 19.7--44.7%, *N *= 8; median test, χ^2 ^= 30.2077, p < 0.0001, d.f. = 4; Figure 2). Straight-chain alkanes *n*-pentacosane (C25) and *n*-heptacosane (C27) comprise the majority of the fertility-related compounds on the eggs of workers and incipient queens.

Although it is not yet known which of the fertility-related hydrocarbons *C. floridanus *workers use to distinguish eggs laid by established queens from eggs laid by incipient queens and workers, 3-methylheptacosane is the most prominent of the fertility-related hydrocarbons in the profile of an established-queen-laid egg. Another 3-methyl alkane, 3-methylhentricontane, was recently identified as the queen fertility pheromone in the ant *Lasius niger *[44], and 3-methyl alkanes correlate with fertility in a number of species [12,51,58-60]. The median percentage of 3-methylheptacosane was greatest for eggs laid by the queen of large experimental colonies (median = 10.9%, range = 5.9--14.7%, *N *= 13), followed by eggs laid by established-queen-laid-egg donors (median = 9.0%, range = 0.0--13.2%, *N *= 15), queens of intermediate experimental colonies (median = 3.2%, range = 0.0--12.9%, *N *= 12), queens of small experimental colonies (median = 0.0%, range = 0.0--0.9%, *N *= 8), and workers (median = 0.0%, range = 0.0--0.0%, *N *= 8; median test, χ^2 ^= 41.9077, *p *< 0.0001, d.f. = 4; Figure 2).

To show that workers from small colonies can perceive and respond to complex hydrocarbon blends, we conducted nestmate recognition bioassays within a week of the egg-discrimination bioassay. Cuticular hydrocarbons are used to discriminate nestmates from non-nestmates [61-63]. In *C. floridanus*, the difference between hydrocarbons of workers from different colonies is much more subtle than the difference in the surface hydrocarbons between eggs laid by individuals of high (i.e., established queens) and low fertility (i.e., workers and incipient queens) [57]. We thus reasoned that ants that can detect the subtle differences between nestmates and non-nestmates can also detect the dramatic differences between eggs laid by workers and established queens [19]. Workers from small colonies were highly effective at recognizing and attacking non-nestmates. In 15 replicates, ants never attacked their own nestmate, but they attacked workers from foreign colonies of the same size in all 15 trials and workers from large foreign colonies in 14 out of 15 trials (Cochran Q test, *Q *= 28.13, *p *< 0.0001).

**Figure 3:**
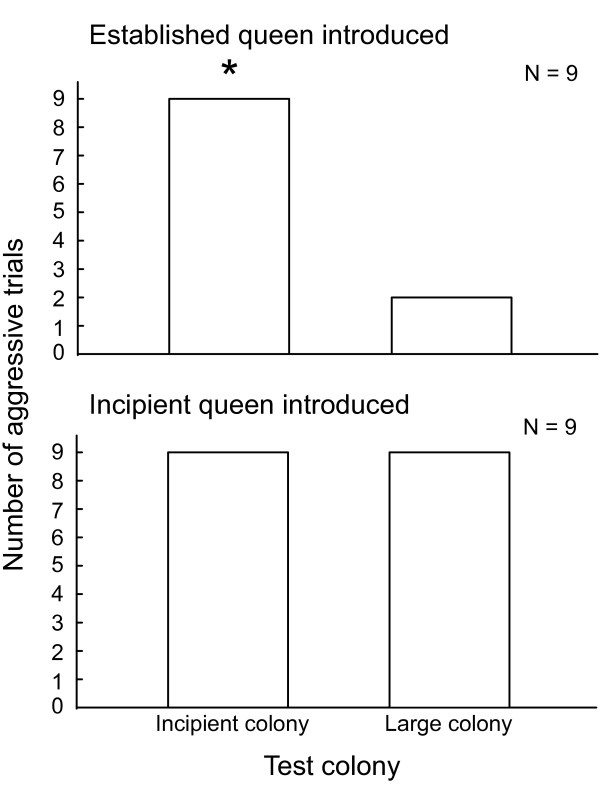
**Number of aggressive outcomes in queen introductions**. Workers from incipient colonies were aggressive toward foreign, established queens significantly more frequently than workers from large colonies. McNemar's test, *z *= 2.64, *p *= 0.008. Workers from both incipient and large colonies were aggressive toward foreign, incipient queens.

In a previous study, we showed workers from established colonies tolerate established queens from foreign colonies [57]. Tolerance of established queens is thought to occur because the queen's fertility overrides information regarding colony membership. To test if workers from incipient colonies responded to fertility information on established queens in the same manner as workers from large colonies, we presented established queens from large colonies (>1000 workers) to workers from incipient colonies (< 40 workers, *N *= 9). The same queens were also introduced to workers from large colonies as a control (*N *= 9). Workers from incipient colonies were highly aggressive toward foreign, established queens. Workers from incipient colonies attacked foreign, high-fertility queens in all 9 replicates. In contrast, workers from large colonies were rarely aggressive toward foreign, high-fertility queens, attacking the introduced queen in only 2 of the 9 trials (McNemar's test, *z *= 2.64, *p *= 0.008; Figure 3). We then performed the study using foreign queens from incipient colonies. Workers from both incipient and large colonies were highly aggressive to foreign, incipient queens. Foreign, incipient queens were attacked in all 9 introductions to workers from incipient colonies and in all 9 introductions to workers from large colonies. In both treatments, queens were attacked significantly more often than expected by chance (binomial test, p < 0.002 for each case).

## Discussion

Our study investigates worker policing behaviour across colony development and addresses both the proximate and ultimate causes of worker policing in social insect colonies. We find a dramatic change in the response to worker-laid eggs between workers from incipient and established colonies. Specifically, we find workers from incipient colonies do not police worker-laid eggs; egg-policing behaviour emerges only when colonies grew sufficiently large (Figure 1). At the ultimate level, our results suggest that the level of conflict within a colony changes across colony development, despite constant relatedness. At the proximate level, our results show the response to fertility information is facultative and changes across colony development.

Our results underscore the importance of factors besides relatedness as the ultimate explanation for patterns of policing behaviour. Relatedness is constant across colony growth in *C. floridanus*, but policing behaviour is absent in incipient colonies and present in large colonies (Figure 1). Relatedness alone cannot account for the pattern of policing behaviour observed in *C. floridanus*.

Mutual worker policing can also evolve when the costs of worker reproduction are high [32,33]. Surprisingly, our results show worker policing is absent in incipient colonies, when the costs of worker reproduction are greatest [6]. Nevertheless, worker reproduction seems not to occur in incipient colonies, since males are not produced and workers do not have developed ovaries (Moore, unpublished data). Why is egg policing unnecessary in incipient colonies? While we cannot exclude coercion by alternate mechanisms, we hypothesize policing behaviour is not expressed in incipient colonies because the costs of worker reproduction are so high that there is no incentive for workers to reproduce [5]. Worker reproduction diverts resources to reproduction at the expense of somatic growth [4,32,33,36-38], and incipient colonies are especially vulnerable to failure [39]. The number of sexuals that can be produced by an incipient colony is negligible compared to the number of sexuals that can be produced if the colony survives to maturity. It is in the workers' self-interest to channel their efforts toward colony growth (i.e., future reproduction) rather than divert resources for immediate male production. Mechanisms for enforcing sterility, including egg policing, are unnecessary if worker sterility is voluntary. Early in colony development, cooperation can occur without external enforcement.

A second, non-mutually exclusive hypothesis for the absence of worker policing in incipient colonies is the cost of recognition errors or informational constraints [64,65]. As our chemical data indicate, eggs laid by workers are very similar to eggs laid by incipient queens in the composition of their surface hydrocarbons, which may be an information constraint or provide insufficient information for a sufficiently large number of correct decisions (Figure 2). To avoid cannibalizing the queen's eggs, workers may be selected to have permissive acceptance thresholds for conspecific eggs in incipient colonies. This is consistent with the general egg tolerance we observed in incipient colonies (Figure 1). In a previous study, we showed that physical policing of reproductive workers is absent in larger colonies of *C. floridanus*[38]. The lack of informational differences potentially encoded in cuticular hydrocarbons between reproductive and non-reproductive workers may be involved here as well.

Our results show that the response of a worker to a conspecific egg changes with the developmental stage of the worker's colony. Workers from incipient colonies tolerated all conspecific eggs, regardless of origin, whereas workers from large colonies destroyed worker-laid eggs. Egg destruction by workers from large colonies is mediated by fertility-related hydrocarbons on the egg surface [19]; workers from large colonies destroy eggs laid by incipient queens and workers, which lack the shorter-chained, fertility-related hydrocarbons found on the surface of eggs laid by established queens [19,52]. Our results show the absence of fertility-related hydrocarbons does not trigger egg-policing behaviour in workers from incipient colonies. The response of workers to fertility information on eggs changes with colony growth.

Interestingly, workers from the largest colony size class tested in the present study showed a trend toward the destruction of eggs laid by foreign, established queens (Figure 1). Although the trend was non-significant, it seemingly conflicts with earlier studies, which show high survival of eggs laid by foreign, established queens [19,52]. One potential explanation for the discrepancy between the current results and those reported previously is that the source colony of the established, queen-laid eggs used in the current study was 1 to 6 years older than the discriminator colony. In previous experiments, the source colony was the same age or younger than the discriminator colony. Our chemical data indicate the median abundance of fertility-related hydrocarbons on the eggs of the older queens was less than the median abundance of fertility-related hydrocarbons on the eggs of yearling queens (Figure 2), potentially as a consequence of limited growth under laboratory conditions [48]. Workers from 1-year-old colonies may have destroyed eggs from older queens when the strength of the foreign queen's fertility signal was weaker than that of their own queen.

Workers from incipient and established colonies also differed in their response to fertility information in the context of queen introductions. Workers from incipient colonies attacked foreign, established queens in every trial, whereas workers from large colonies tolerated foreign, established queens in all but two trials (Figure 3). Workers from both incipient and large colonies attacked foreign, incipient queens (Figure 3). Tolerance of foreign, established queens by workers from large colonies is thought to occur because the queen's fertility status overrides colony membership information [57]. Incipient queens lack a strong fertility signal, and thus workers from both incipient and large colonies attack them. The rejection of established queens by workers from incipient colonies shows that a strong fertility signal does not guarantee acceptance in every context. It also contrasts with our findings from the egg-policing assay, in which workers from incipient colonies tolerated eggs laid by foreign, established queens. This indicates a worker's response to fertility information depends on the recognition context: eggs or adults. Together, our egg-policing and queen-tolerance assays demonstrate the response to fertility information is not fixed, but changes across colony development.

This paper is the first to show that a worker's response to fertility signals changes with colony life stage, but we expect it is a widespread phenomenon. If queen fertility pheromones indeed serve as an honest indication of reproductive capacity [40,49], then changes in fertility signals corresponding with colony size should be a common feature of social insect colonies. When the queen's fertility signal changes throughout colony development, we predict workers' response to fertility information also changes [48].

Further research is necessary to identify the proximate mechanisms that account for the change in workers' responses to fertility signals. The change in behaviour may be the result of physiological differences between workers from incipient and large colonies (e.g., maternal effects), or the behaviour may be triggered by environmental cues. In particular, experience with fertility signals may change workers' acceptance thresholds [48,66,67]. It is also possible that the egg-tolerance observed in small colonies is due to age-dependent expression of policing behaviour; for example, if ants do not show policing behaviour until they reach a certain age, then egg policing may be absent from small colonies because the workers are not sufficiently old to demonstrate policing behaviour [34,68]. Similarly, if worker sterility in incipient colonies is voluntary, then it is necessary to determine the mechanism that induces worker sterility in incipient colonies. In large colonies, queen-laid eggs with fertility-related hydrocarbons have been shown to induce worker sterility [19], but no such eggs exist in incipient colonies. Determining the proximate mechanisms that generate the behavioural change we report here is critical to understanding the regulation of reproduction across colony development.

Although the regulation of worker reproduction has been studied intensively in social insects, very few studies have investigated the regulation of worker reproduction across the colony life cycle. This is problematic at two levels. First, the proximate mechanisms regulating worker reproduction can change across colony ontogeny [69], and these changes will only be apparent in studies that consider a range of developmental stages. Second, the intensity of regulation may change during colony development, according to the ultimate explanations for the evolution of policing behaviour. Ohtsuki & Tsuji were the first to predict that the level of policing behaviour in a colony depends on the colony's developmental stage [6]. Specifically, their model predicts policing behaviour will be expressed in growing colonies because male production early in the colony life cycle reduces the future inclusive fitness of colony members. Our results do not match this prediction; we find workers from incipient colonies do not destroy worker-laid eggs, and policing behaviour emerges only with colony growth. However, our results are consistent with the broader prediction of Ohtsuki & Tsuji's model, which is that the expression of policing behaviour depends on the stage of colony growth. Perhaps the absence of egg policing in very small colonies, such as the incipient colonies tested here, represents an unanticipated phase of colony growth in which worker sterility is self-imposed and policing is not necessary. It is also possible that the self-restraint of workers from incipient colonies is the result of strong policing in the past, which has subsequently resulted in reproductive acquiescence in *C. floridanus *workers from small colonies.

Another prediction of Ohtsuki & Tsuji's model is that worker policing should subside in monogynous, monandrous species once the colony reaches reproductive maturity [6]. The largest colonies tested in the current study contained only 1000 to 2000 workers, whereas field colonies can grow up to 10,000 workers in size. Just as our current results show that the behaviour of a colony with 80 workers cannot be extrapolated from the behaviour of a colony with 1000 workers, the behaviour of a colony with 10,000 workers cannot be extrapolated from the behaviour of a colony one-tenth its size. To understand the ultimate causes of social regulations in social insects, it is essential to test regulatory behaviour across the life cycle of the colony.

## Conclusions

The response to fertility information in an ant colony changes radically during colony ontogeny. We found workers from incipient colonies tolerate all conspecific eggs, but are aggressive toward foreign, established queens. In contrast, workers from large colonies discriminate against worker-laid eggs, but tolerate foreign, established queens. Together, these results show the response of workers to the presence or absence of fertility information changes over the course of a colony's life cycle. At the ultimate level, our results stress the importance of factors other than relatedness for understanding the regulation of reproduction. In particular, we suggest levels of intracolonial conflict change across colony development, and as a consequence, mechanisms for managing conflict also change.

Nearly everything we know about social regulation in social insects comes from colonies beyond the earliest phases of colony growth. This study shows that regulatory mechanisms observed in one stage of colony development cannot be generalized to all stages of a social insect colony's life cycle. We are missing critical parts of the colony's life cycle in our understanding of social regulation of reproduction. Further research is necessary to understand how social insect colonies are organized across the colony life cycle.

## Methods

### Animals and culturing conditions

Founding queens were collected after mating flights in the Florida Keys, USA, in August 2001, July 2002, November 2006, August and October 2007, August 2008 and August and November 2009. The queens were transferred to the lab and cultured as described in [19,52,57]. Queens were cultured singly because *C. floridanus *is haplometrotic.

### Egg discrimination bioassay

To determine how egg-policing behaviour changes with colony size, we tested the egg-policing behaviour of workers from 15 colonies collected in August and October 2007 at three points during the colony's development: when they contained 60 to 80 workers (small colony size), 200 to 300 workers (intermediate colony size) and more than a 1000 workers (large colony size). These sizes correspond to groups B, C, and D in [52] and were reached 4 to 6, 7 to 9, and 10 to 14 months after being collected as foundations in the field, respectively. At each size, we tested the response of workers to eggs laid by their own queen; eggs laid by a non-nestmate, established queen; and to eggs laid by non-sister workers. Three groups of twenty ants each were isolated from the experimental colonies with water and sugar-water and allowed to habituate for 30 minutes before receiving 10 eggs from one of the three egg sources. After 24 hours, we counted the number of eggs remaining. The study was not performed blind, but because *C. floridanus *eggs are relatively large (0.1 cm) and easily visible to the naked eye, the egg counts should be robust to observer bias. Eggs laid by non-nestmate, established queens came from large (>1000 workers), healthy colonies collected in August 2001, July 2002, and November 2006. Worker-laid eggs came from non-sister workers in worker groups originating from colonies collected in August 2001 and July 2002 and orphaned 4 to 18 months before testing. Due to logistical limitations, worker-laid eggs and established-queen-laid eggs for our egg discrimination bioassay had to come from non-nestmates. In other ant species, workers discriminate against eggs originating from non-nestmates [67]. However, two lines of evidence suggest the egg-layer's colony membership is less important than the egg-layer's fertility status in determining a worker's response to an egg in *C. floridanus*: (1) workers from large colonies destroy eggs laid by sister workers [19] and (2) workers from large colonies do not destroy eggs laid by foreign, established queens [19,52].

#### Statistical analysis

Due to extreme heteroscedasticity of the egg survival data, parametric approaches were not appropriate. We used a randomization analysis of a multifactor ANOVA with colony size and egg source as fixed factors and recipient colony as a random factor; we used 10,000 rearrangements of the data to calculate the p-value [70]. Randomization analyses are robust to heteroscedasticity when sample sizes are equal [71]. Analysis was done in SAS (SAS Institute, Inc.) with the randomization wrapper written by Cassell [72]. A randomization analysis of a paired t-test was used for post-hoc comparisons. P-values calculated by randomization analyses are denoted by a subscript indicating the number of permutations.

### Chemical analysis

We analyzed the egg surface hydrocarbons of a subset of the eggs laid by workers (*N *= 8) and by foreign, established queens (*N *= 15), as well as eggs laid by the experimental colony queens when their colonies were small (*N *= 8), intermediate (*N *= 12), and large (*N *= 13) in size. Sample sizes reflect the number of colonies from which we removed 10 eggs and extracted the eggs' surface hydrocarbons in 100 μl of hexane for 2 minutes. The hexane was transferred to a clean vial and allowed to evaporate. We reconstituted the extract in 5 μl of hexane and injected 1 μl of the resulting suspension in the injection port of an Agilent 6980N series gas chromatograph (GC). Further details are described in [19]. Peak areas were measured in Enhanced ChemStation (Agilent Technologies 2005). We divided the egg surface hydrocarbon profiles into two parts as in [52]: the shorter-chained compounds characteristic of high-fertility queen eggs (*n*-pentacosane to 10-methyl-, 12-methyl, and 14-methyloctacosane), and the longer-chained compounds common to all eggs (12,16-dimethyloctacosane to 5,9,13,17-tetramethyltritriacontane) [19,52,57]. We summed the peak areas of each part of the profile and compared the proportion of the overall profile represented by the shorter-chained fertility hydrocarbons using the median test in Statistica 7.1 (StatSoft Inc.). Two-sample median tests were used for the post-hoc comparisons; the Bonferroni correction was applied to account for the number of comparisons. We analyzed the proportion of the entire hydrocarbon profile represented by 3-methylheptacosane, the most prominent compound in the fertility signal, using the same approach. The 3-methylheptacosane peak of one exceptional established queen was below the determined detection threshold.

### Nestmate recognition bioassay

**Figure 4:**
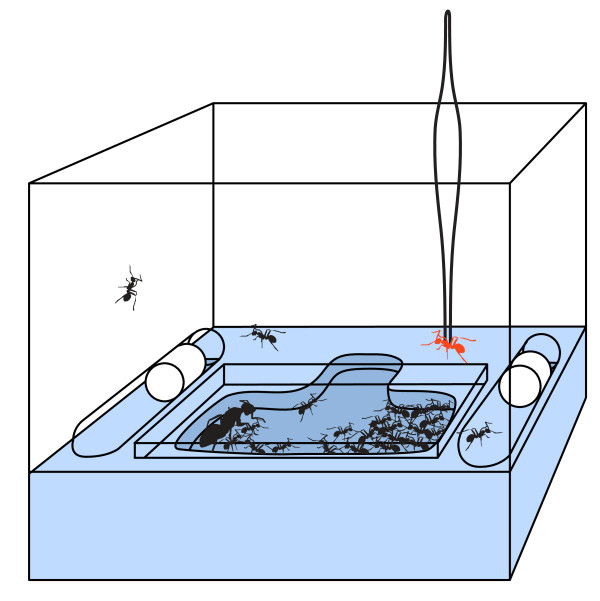
**Experimental set-up for nestmate recognition assay**. For the nestmate recognition assay, focal ants were introduced directly into the incipient colony's nest box. Each colony is housed in a transparent plastic box (10 cm wide × 7 cm high) with a dental plaster floor and a molded nest chamber. The nest chamber is covered with a plate of glass and has only one entrance. In incipient colonies, nearly all the workers are in the nest chamber; very few workers are outside the nest. With clean forceps, we lowered the focal ant (shown in red) into an unoccupied area of the nest box and the observed the reaction of the colony members to the introduced ant for 5 minutes.

We tested the reaction of the ants in the small colonies to individuals from their own colony, individuals from another small colony (60 to 80 workers), and individuals from a large colony (>1000 workers) [73]. The introduced ants were removed from their colony and painted with a single dot of white Testor's enamel paint and allowed to dry for one hour. Meanwhile, we opened the lid of the experimental colony and allowed the ants to settle for at least 20 minutes. We then gently lowered one of the introduced ants into the nestbox with clean forceps (Figure 4). We observed the reaction of the experimental ants to the introduced ants for 5 minutes or until aggression was observed. An introduction was classified as "aggressive" if the experimental ants bit and held the focal ant or sprayed it with formic acid. The order of the introductions was random and the experimenter was blind to the identities of the individuals. We analyzed the aggression data using Cochran's Q test.

### Tolerance of foreign queens by workers from incipient colonies

We tested the response of workers from incipient colonies (< 40 workers, *N *= 9) to established queens from large colonies (>1000 workers). The same queens were also introduced to workers from large colonies as a control. The order of introductions for each queen (small or large colony first) was random, and we waited at least 24 hours between introductions. The small colonies were reared from foundresses that had been collected in August 2008 and the large colonies were reared from foundresses collected in August and September of 2007.

Twenty workers from the experimental colony were removed and isolated in a circular arena (8.4 cm diameter × 3.5 cm height) lined with clean copy paper. The arena's wall was coated with Fluon (Northern Products Inc.) to prevent escape. The workers were allowed to habituate for 30 minutes before the focal queen was gently lowered into the arena using clean forceps. The queen had been removed from her own colony and isolated with 5 worker ants at least 30 minutes before the start of the trial.

The queen remained in the arena for 3 minutes after its first encounter with one of the experimental workers or until aggression was observed. If aggression was observed, the queen was removed immediately from the arena to minimize the damage inflicted by the workers. An introduction was classified as "aggressive" if the experimental ants bit and held the focal ant or sprayed her with formic acid. All introductions were recorded on HDV film. We analyzed the aggression data using McNemar's test.

The same procedure was used to contrast the response of workers from incipient and large colonies to foreign, incipient queens. Incipient queens and workers came from colonies collected in August and November 2009. Incipient colonies had 28 to 178 workers at the time of testing (median = 86, *N *= 9). Workers from large colonies came from colonies collected in August 2008.

## Authors' contributions

DM and JL designed the experiments and wrote the manuscript. DM conducted the experiments and statistical analyses. All authors read and approved the final draft.
